# Multiapproach Analysis Combined with Chemometrics for the Authentication of Commercial Oils of *Croton tiglium* (L.)

**DOI:** 10.1021/acsomega.5c09251

**Published:** 2025-10-30

**Authors:** Anna Claudia M. O. Capote, Patrícia M. Campos, Wilmer H. Perera, Airton Kist, Wendy K. Strangman, Thomas Williamson, Sarah A. Barr, Carlos G. Wambier, Flávio L. Beltrame

**Affiliations:** † Pharmaceutical Science Post-Graduation Program, 67883 State University of Ponta Grossa, Ponta Grossa, Parana 84030-900, Brazil; ‡ Department of Pharmaceutical Sciences, State University of Ponta Grossa, Ponta Grossa, Parana 84030-900, Brazil; § CAMAG Scientific Inc., Wilmington, North Carolina 28401, United States; ∥ Department of Mathematics and Statistics, State University of Ponta Grossa, Ponta Grossa, Parana 84030-900, Brazil; ⊥ Department of Chemistry and Biochemistry, 14621 University of North Carolina Wilmington, Wilmington, North Carolina 28403-3201, United States; # Department of Dermatology, The Warren Alpert Medical School of Brown University, Providence, Rhode Island 02903, United States

## Abstract

**Background:** *Croton tiglium* (L.) seed oil (CO) is the main component of a formula used in deep chemical peeling at dermatologist offices around the world. The phorbol esters present in CO are responsible for pro-inflammatory effects and new collagen production. However, concerns have been raised about the authenticity of commercially available oils labeled and sold as CO, due to the potential lack of quality in many products resulting from the challenging extraction process and high production costs. **Objective:** This study proposed a multiapproach quality control analysis combined with chemometrics evaluation to verify the authenticity of commercial CO products. **Methods:** As an initial screening step, organoleptic analyses of 10 commercial CO samples were performed to assess visual characteristics followed by nuclear magnetic resonance, high-performance thin-layer chromatography, and liquid chromatography-mass spectrometry techniques to evaluate the chemical profiles and chemometrics analysis to classify and corroborate the authenticity of the evaluated samples. **Results:** Three samples failed to exhibit the standard organoleptic characteristics and CO chemical fingerprint, while one showed a low phorbol ester (chemical marker) concentration. Chemometric analysis allowed the discrimination of sample groups based on their chemical similarity, highlighting its effectiveness in distinguishing authenticity from potential adulterated or nonstandard products. **Conclusion:** The analysis demonstrated that the association of the proposed techniques together with chemometric data can ensure the authenticity of the CO commercial products used in dermatological areas as the active component on deep peeling formulas and can be used as a protocol for evaluation of CO commercial products by suppliers or industries.

## Graphical abstract

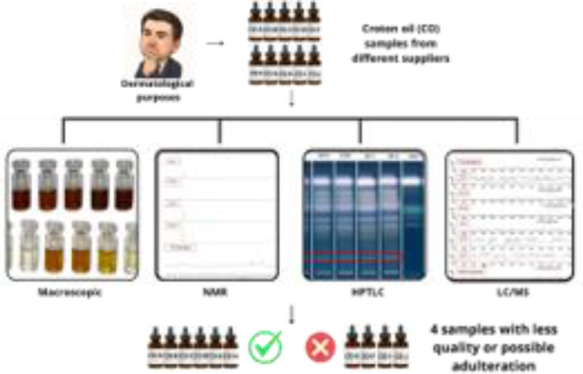

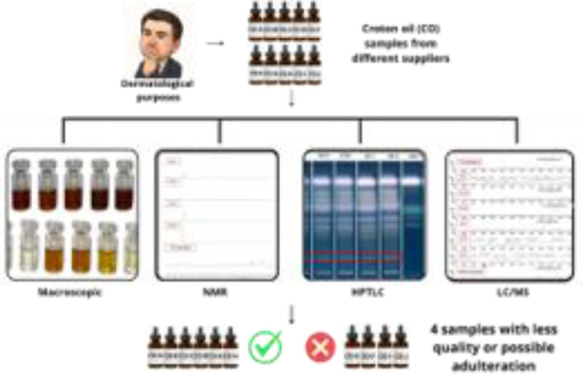

## Introduction

*Croton tiglium* L. oil (CO) is a viscous liquid obtained from the seeds of an Asiatic tree belonging to the spurge family, Euphorbiaceae. The biological activities of CO described in the literature are related to antioxidant, antimicrobial, anti-inflammatory, neuroprotective, antitumor, anticancer, cytotoxic effects and are largely attributed to its high content of fatty acids, triglycerides, and particularly phorbol esters.^1,2^ These constituents form the basis for both traditional medicinal uses and contemporary therapeutic applications. The use of CO as a purgative medicinal substance was introduced to the west from China by the Dutch in the 16th century. In the 19th century, the physician (and pioneer in the field of signal transduction) Sidney Ringer described its topical application in the treatment of ringworm (external irritant, being powerful when applied upon the face, scalp, larynx, and chest), and over time, this plant-derived matrix was recognized for its dermatological applications.^3^

Currently, CO is an active matrix prevalent in dermatology offices because of its diverse and remarkable properties, such as promoting epidermal exfoliation and dermal collagen production. When used in deep chemical peeling formulas, particularly in antiaging treatments, it can promote the skin’s overall structure in addition to improving its external appearance.^4,5^

The dermatological therapeutic efficacy of CO is related to the presence of secondary metabolites, described as tigliane-type diterpenes, like phorbol 12-myristate 13-acetate (PMA), that can activate protein kinase C (PKC). Upon binding to the C_1_ domain of PKC, these esters mimic the natural ligand diacylglycerol, triggering the sustained activation of PKC isoforms. This persistent PKC activation initiates a complex pro inflammatory cascade, leading to an upregulation of transcription factors, and stimulating the production of various pro-inflammatory cytokines, growth factors, and matrix remodeling enzymes. In a topical application this can result in skin rejuvenation promotion by stimulating fibroblast activity, promoting collagen production and dermal remodeling, and enhancing skin texture.^4,6,7^

Through this mechanism, CO has gained popularity in various cosmetic and dermatological treatments. Since the 1960s, CO has been utilized in combination with phenol in a homemade dermatologic formula, for deep chemical peeling procedures, an approach initially developed by Baker & Gordon and later refined by Hetter. This treatment has been permanent and massively utilized due to these prominent antiaging effects in dermatology, delivering significant enhancements in skin rejuvenation of patients.^5,8^ However, achieving these therapeutic benefits depends on the optimal concentration and composition of phorbol esters in CO, since higher CO concentration is directly associated with greater skin permeation and a more pronounced final effect.^9,10^

Despite the efficacy of CO, concerns about the authenticity and purity of this material persist in the medical field due to the presence of low-cost, nonedible oils, or even cooking oils introduced as adulterants to reduce costs. This risk of adulteration is not uncommon among valuable natural substances, making it crucial to establish robust methods for authenticating natural matrices, such as CO and detecting any potential adulteration.^11^

Several analytical techniques, including high-performance thin-layer chromatography (HPTLC), nuclear magnetic resonance (NMR), and liquid chromatography-mass spectrometry (LC-MS), are available for the detection of adulterants and the authentication of oil and vegetables matrices. NMR is a simple, rapid, nondestructive acquisition method of comprehensive chemical fingerprints, allowing for the collection of large amounts of data and facilitating authentication and adulteration detection without the need for extensive sample preparation.^12,13^ HPTLC serves as a cost-effective, robust, sensitive, and efficient screening technique able to analyze multiple samples simultaneously, generating fingerprints that support preliminary quality assessments.^11,14–16^ LC-MS offers high sensitivity and selectivity for detecting and quantifying minor components and adulterants and able to handle complex mixtures. Together, these techniques enhance the accuracy and robustness of quality control protocols for complex natural products.^17^

The present research applied several orthogonal analytical techniques, including organoleptic evaluation, HPTLC, NMR and LC-MS combined with chemometrics analysis, for the authentication of commercial oils of *Croton tiglium* (L).

## Results and Discussion

According to the literature, the color of CO is described as pale yellow to brown and typically transparent. The odor is characterized as a pungent and unpleasant smell.^3^ This initial step revealed significant color variations among the purchased CO samples, in which three samples (CO-E, CO-F, and CO-J) displayed significant color and odor differences, when compared to the CO standard (Sigma-Aldrich, baseline reference: brown color and pungent smell); some of them resembled the edible oil samples evaluated in this study. Also, the samples CO-G, CO-H, and CO-I presented a light scent and color when compared to the CO standard (Figure 1). Through this macroscopic analysis, preliminary observations showed that organoleptic characteristics are indicators of product suitability or possible adulteration or substitution.

**Figure 1:**
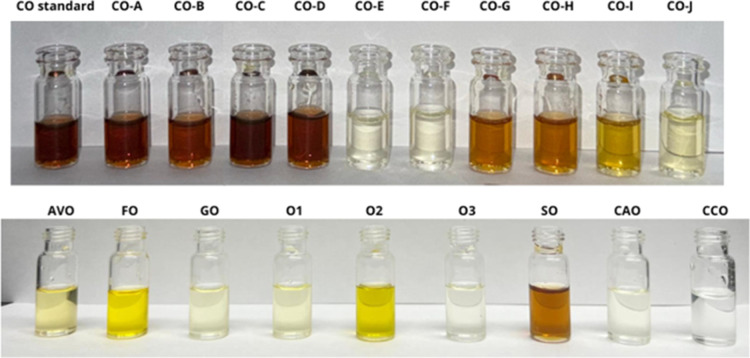
Organoleptic characteristics presentation. CO standard (Sigma-Aldrich standard sample); CO-A, CO-B, CO-C, CO-D, CO-E, CO-F, CO-G, CO-H, CO-I, CO-J (commercial CO samples); AVO (avocado oil); FO (flax oil); GO (grapeseed oil); O1 (olive oil—sample 1); O2 (olive oil—sample 2); O3 (olive oil—sample 3); SO (sesame oil); CAO (canola oil); CCO (coconut oil).

Due to the high cost of CO, it has been reported that other types of edible oils are deliberately added to the mixture of these products for the purpose of substitution or adulteration.^14^ Environmental challenges, geopolitical circumstances, and rising costs contribute to the scarcity of raw materials, increasing the risk of adulteration, which is both concerning and unethical.^11,13^

After organoleptic analysis, we next turned to NMR profiling of the commercial CO samples; NMR data were acquired, and the major resonances observed in these vegetal matrices were determined^12,13,18,19^ (Table 1).

**Table 1:** ^1^H NMR Data for 5 Major Resonances in the Observed ^1^H NMR Spectra

signal	chemical shift (ppm)	chemical group
A	1.40–1.15	(−(CH_2_)_*n*_−)
B	1.70–1.50	(−OCO–CH_2_–CH_2_−)
C	2.80–2.70	(=HC–CH_2_–CH=)
D	∼5.30	>CHOCOR
E	∼5.40	(−CH=CH−)

In the tested commercial CO products, PMA concentrations are below the limit of detection for the sample concentration available. The five major resonances listed in Table 1 correspond to chemical shifts of unsaturated fatty acids used to differentiate the samples (Figure S1—^1^H NMR spectra). These signals were used to generate principal component analysis (PCA) of commercial CO products. The PCA strategy is a multivariate statistical technique that reduces the dimensionality of complex data sets while preserving the variation present, allowing for visualization of patterns and chemical differences among samples that are consistent with analytical measurements. For this reason, chemometric techniques, such as PCA, were employed to investigate differences among the samples analyzed. This analysis revealed that CO-E, CO-F, CO-I, and CO-J exhibited spectra profiles distinct from the CO standard and from CO-A, CO-B, CO-C, CO-D, CO-G, and CO-H (Figure 2A). Chemometric analysis of the edible oil samples and CO-E, CO-F, and CO-I revealed that these four commercial CO samples present similarities with most notably coconut oil and olive oil (clustered closely) (Figure 2B).

**Figure 2:**
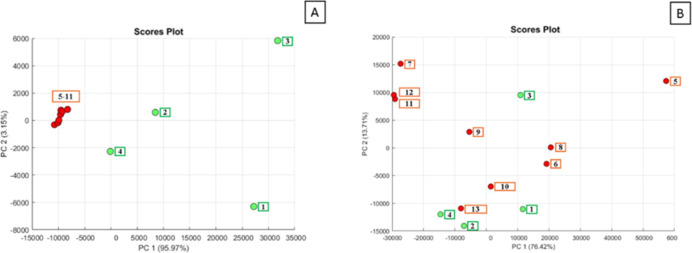
NMR data. (A) Chemometric assay comparison CO standard and commercial samples: green cluster—1: CO-E, 2: CO-F, 3: CO-I and 4: CO-J.; red cluster—5: CO standard, 6: CO-A, 7: CO-B, 8: CO-C, 9: CO-D, 10: CO-G and 11: C–H. (B) Chemometric assay comparison of edible oils samples and CO commercial samples: green cluster—1: CO-E, 2: CO-F, 3: CO-I and 4: CO-J.; red cluster—edible oils samples (5: AVO, 6: CAO, 7: CCO, 8: FO, 9: GO, 10: O1, 11: O2, 12: O3, 13: SO).

As the phorbol esters were not easily detectable in the ^1^H NMR spectra of COs due to the naturally low concentrations, a modern planar chromatographic method was developed to evaluate the commercial CO samples based on their chemical fingerprint. To show specificity, the analysis included the CO standard sample along with various edible oils commonly used as substitutes or adulterants. One distinctive chemical marker, PMA, a representative tigliane-type diterpene (phorbol esters) present in CO, was also used^20^ (Figure 3). The PMA band (track 2), with a *R*_f_ value of 0.15, is clearly visible in the CO standard sample (track 3). Several other bands with different colors and intensities, mainly below *R*_f_ 0.4, also help differentiate this vegetal matrix from the edible oil samples (track 4–12). Edible oils were selected as potential adulterants due to their chemical similarity to CO, particularly regarding their fatty acid composition. Their wide availability and low cost make them plausible substitutes in adulteration practices, justifying their inclusion in the comparative HPTLC analysis. Bands with *R*_f_ values above 0.4, observed in all samples, are likely associated with fatty acids commonly present in vegetal matrices and are observed as the dominant compounds in the NMR spectra.

**Figure 3:**
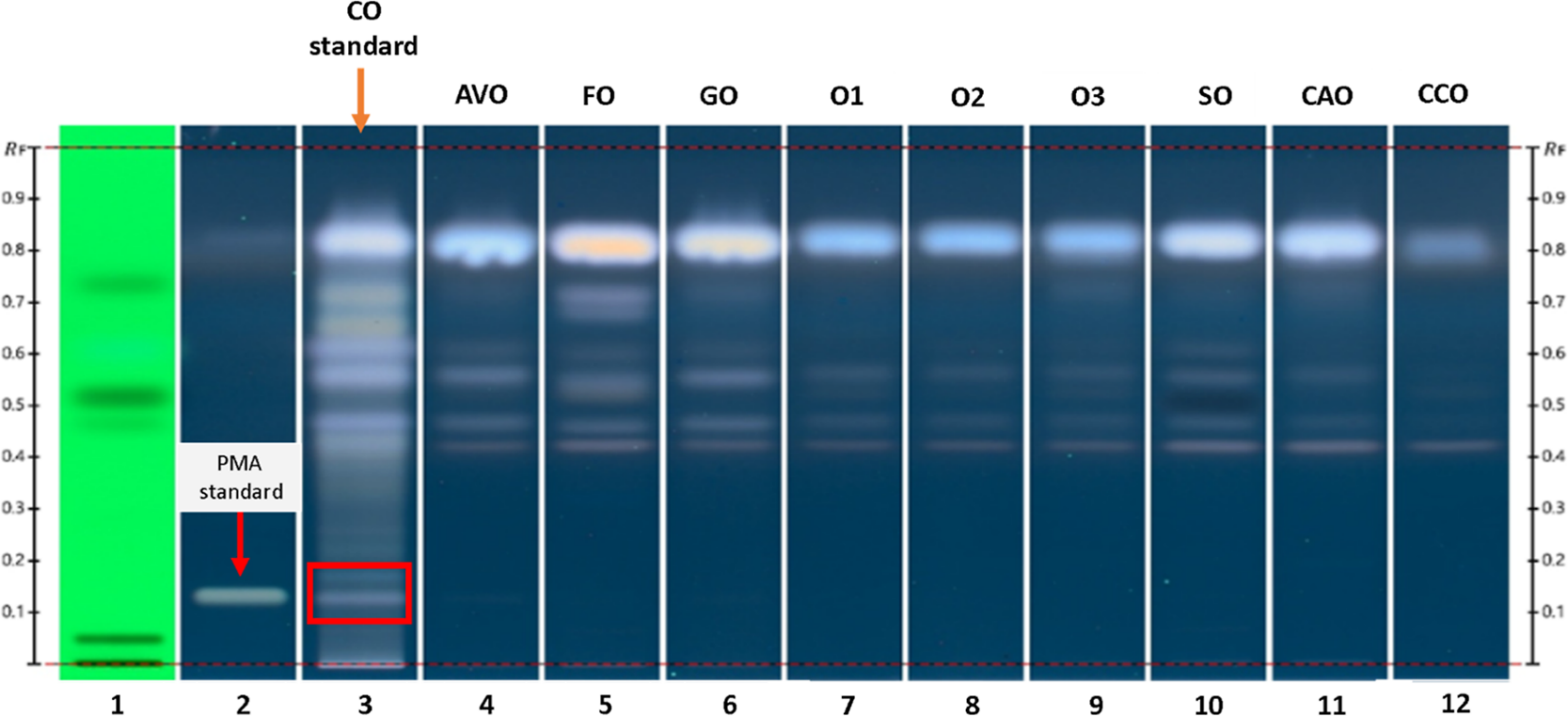
HPTLC profiles of the system suitability test under shortwave UV (track 1), PMA standard (track 2), CO standard (track 3), avocado oil (track 4) flax oil, (track 5), grapeseed oil (track 6), olive oil 1 (track 7), olive oil 2 (track 8), olive oil 3 (track 9), sesame oil (track 10), canola oil (track 11), coconut oil (track 12). The red rectangle indicates the *R*_f_ position related to PMA.

The HPTLC method was fully validated, and the system suitability test (SST) (track 1) showed quenching zones at *R*_f_ 0.05 ± 0.01 for paracetamol; *R*_f_ 0.55 ± 0.02 for 9-hydroxyfluorene; and *R*_f_ 0.76 ± 0.02 for 2-(2*H*-benzotriazol-2-yl)-4-(1,1,3,3-tetramethylbutyl) phenol. The SST was performed prior to and during HPTLC analysis to ensure the reliability and reproducibility of the method. This analysis confirmed that the plates, mobile phase, and detection system were functioning properly, allowing accurate comparison of CO samples.

For this feasibility study, 10 commercial CO samples and their chemical fingerprints were compared to the CO standard sample, as shown in Figure 4. Of the 10 commercial CO samples analyzed, CO-E, CO-F, and CO-J did not exhibit a chemical profile like that of the CO standard, particularly regarding the PMA band at *R*_f_ 0.15, which was absent, indicating that these commercial CO samples cannot be identified as croton oil. Samples CO-A, CO-B, and CO-D showed a similar fingerprint to the CO standard sample, while samples CO-C, CO-G, and CO-H were identified as CÓs, but additional intense bands were observed between PMA and *R*_f_ 0.4. Although sample CO-I showed a comparable fingerprint to the CO standard, the intensity of the PMA was fainter than the one in the CO standard, suggesting the presence of a low amount of PMA in this sample (Figure 4). These HPTLC findings are consistent with organoleptic evaluation and NMR analysis.

**Figure 4:**
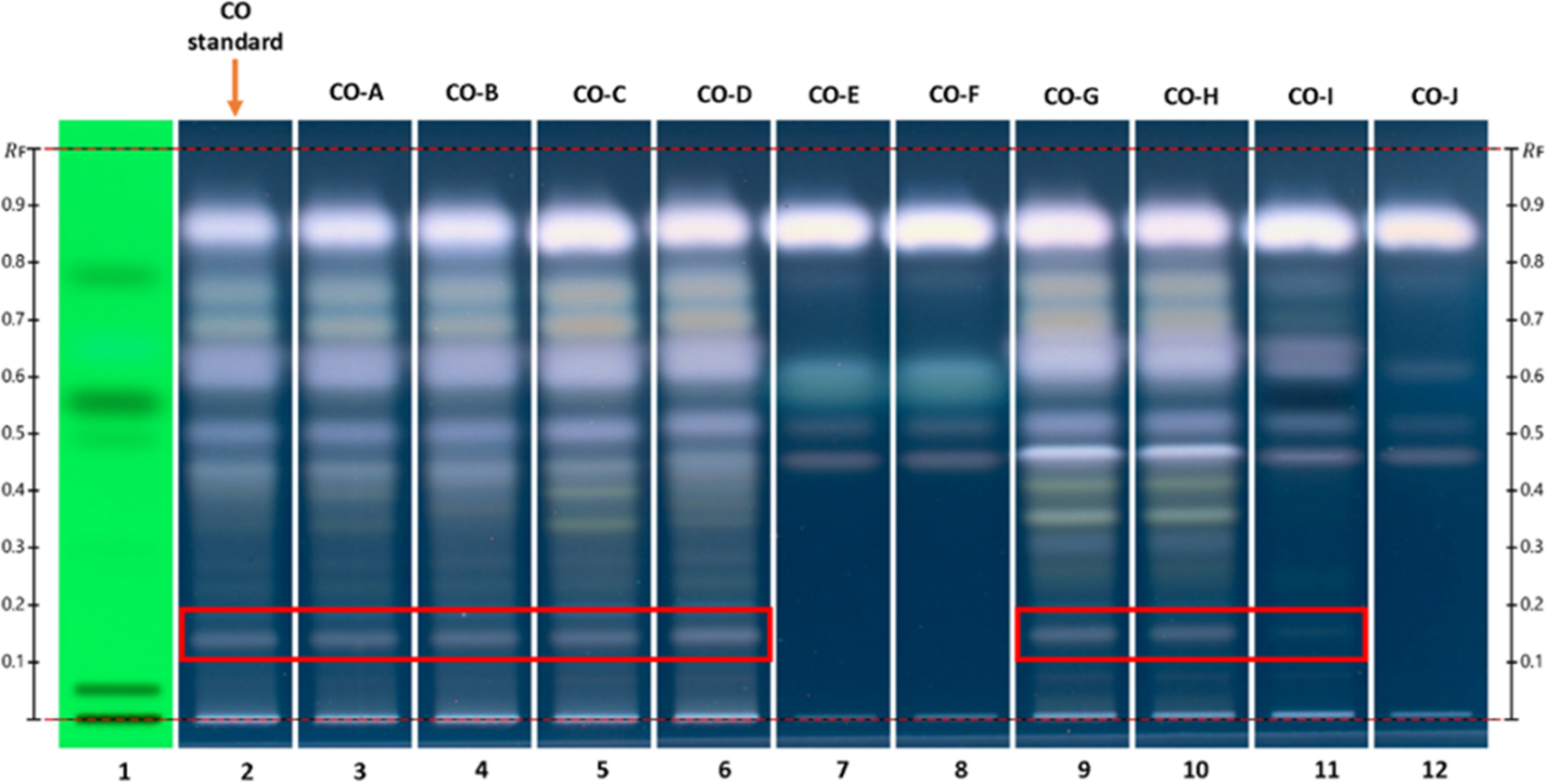
HPTLC plate showing the different chemical profiles obtained from the commercial CO samples visualized under 366 UV light. SST (track 1), CO standard (track 2), CO-A (track 3), CO-B (track 4), CO-C (track 5), CO-D (track 6), CO-E (track 7), CO-F (track 8), CO-G (track 9), CO-H (track 10), CO-I (track 11), CO-J (track 12).

The application of HPTLC methodology is already recognized as reliable, accurate, cost-effective, simple, and important for the investigation of natural products. It can also be used to identify adulterants or evaluate the authenticity and quality of raw materials and finished products.^11^ This type of evaluation has already been conducted involving 20 samples of citronella oil and the HPTLC analysis enabled the identification of two distinct chemotypes, as well as the detection and quantification of triglycerides, indicating possible adulteration of the vegetable oils.^14^ Similarly, in a study on argan oil, HPTLC revealed products containing less than 0.5% argan oil,^15^ demonstrating HPTLC high specificity. Furthermore, a study using TLC on copaiba oil identified three samples adulterated with soybean oil, based on their *R*_f_ values.^16^

HPTLC fingerprinting served as the basis for generating a chemometric PCA plot (Figure 5). These data illustrate how the CO samples cluster according to their chemical composition. In this statistical analysis, samples with similar spectral profiles are positioned closer together, whereas those with distinct profiles are plotted farther apart. This clustering reflects variations in the relative abundance of compounds, such as unsaturated fatty acids and phorbol esters. The graph revealed that the CO standard sample and samples CO-A, CO-B, and CO-C and CO-D clustered closely with two other samples (CO-G and CO-H), indicating a strong similarity in their chemical profiles. Four commercial samples (CO-E, CO-F, CO-I, and CO-J) occupied a central region in the plot, displaying a notable divergence from the CO standard. In addition, all commercial edible oil samples appeared at the opposite end of the chemometric graph and were clearly separated from the CO standard cluster. This spatial distribution suggests no correlation among the commercial edible oils and the CO standard cluster, thereby ruling out the possibility of fraud with these selected oils.

**Figure 5:**
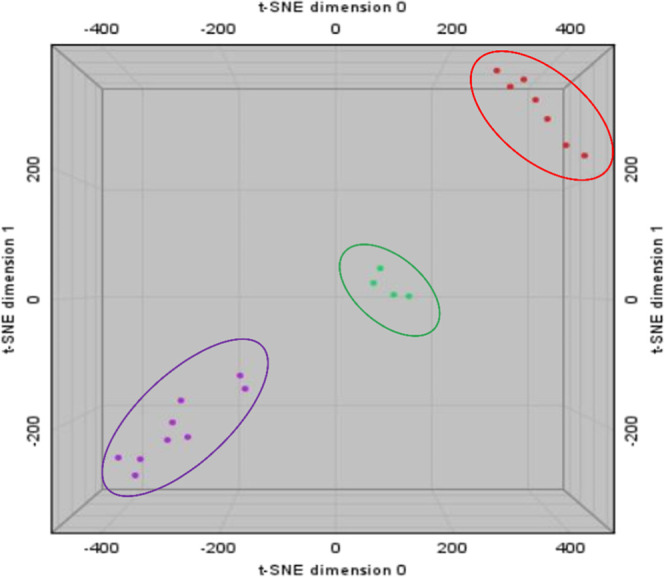
Chemometric assay based on HPTLC analysis. Red cluster: CO standard, CO-A, CO-B, CO-C, CO-D, CO-G, CO-H. Green cluster: CO-E, CO-F, CO-I, CO-J. Purple cluster: AVO, FO, GO, O1, O2, O3, SO, CAO, CCO.

As part of a comprehensive study, orthogonal analysis using LC-MS was performed. The LC-MS technique is already widely employed to detect adulteration in oils. This analytical approach enables the identification of metabolic markers that serve as indicators of sample authenticity. By investigating the chemical composition of oils, this method enables product authentication based on the presence and concentration of specific and intrinsic compounds. In addition to chemical analysis, statistical tools can be applied concurrently to support data interpretation and increase reliability of results.^21,22^ Previous studies on this natural matrix have developed methodologies to identify the markers of authenticity. Eleven metabolic biomarkers associated with the isoflavonoid biosynthesis pathway have been described as reliable indicators for detecting adulteration in CO.^23^ PMA, a secondary metabolite frequently found in CO,^24,25^ serves as a diagnostic marker.

During LC-MS analysis, the molecular ion at *m*/*z* 639.3885 [616.398 + Na]^+^ and three fragment ions at *m*/*z* 411.1716, *m*/*z* 351.1507, *m*/*z* 333.1295 were observed^26^ (Figure 6). This observation clearly aligns with previous results in the HPTLC analysis where the PMA band was observed at *R*_f_ 0.15.

**Figure 6:**
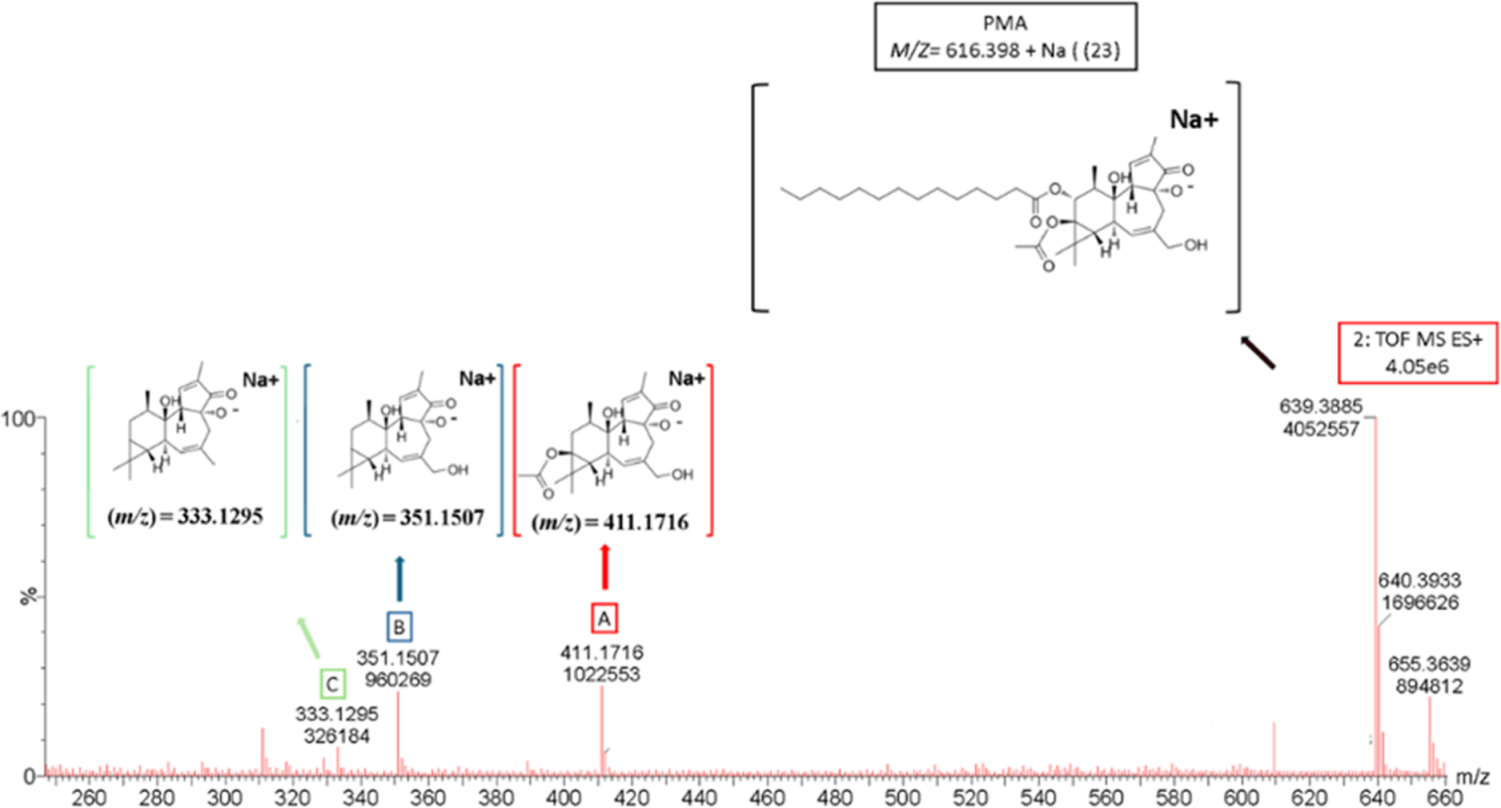
LC-MS spectrogram diagnostic peaks of the PMA standard and fragment ion structures.

All samples were prepared to the same concentration, so integration of extracted ion chromatogram (EIC) data for *m*/*z* 639 peaks, where present, enabled qualitative assessment of relative concentrations of PMA-like compounds within each of the samples and corroborated the HPTLC profiling (Figure S2—LC-MS spectra). Relative integrated ion counts varied considerably between samples (Table 2) and were in agreement with the HPTLC profiles.^21^

**Table 2:** PMA Chromatogram Identification and Relative Quantification

sample	retention time	*m*/*z* [PMA + Na]^+^	extracted ion count
PMA standard	6.24	639.3873	243,026
CO standard	6.24	639.3884	28,168
CO-A	6.25	639.3866	29,588
CO-B	6.22	639.3879	30,406
CO-C	6.24	639.3879	38,820
CO-D	6.24	639.3868	49,184
CO-E			
CO-F			
CO-G	6.27	639.3867	28,496
CO-H	6.24	639.3870	25,556
CO-I	6.24	639.3883	11,284
CO-J			

This methodology has already been used in previous studies to perform detailed chemical profiling and assess the authenticity of various products. One of these studies used LC-MS-based metabolomic profiling to identify specific markers in five types of unrefined, cold-pressed seed. These markers were essential for evaluating the oil authenticity and detecting potential adulteration. By characterizing the unique metabolite compositions of these oils, the research provides a scientific basis for quality control, ensuring product integrity and protecting consumers against fraud. The results also revealed differences in the intensity of oil markers among products from different producers and production batches, shown by EICs and their relative standard deviations.^22^ Relative quantification is widely applied in the analysis of various raw materials, including peptides. In another study, this strategy was carried out by integrating the peak areas of the EIC from high-resolution mass spectra. The results demonstrated that when multiple peptides were analyzed and the data carefully processed, the approach provided high sensitivity, reproducibility, and a broad dynamic range. The use of peptide-level EICs proved to be a reliable and effective method for accurate protein quantification in complex biological samples.^27^

The main finding was the complete absence of PMA in CO-E, CO-F, and CO-J. Additionally, sample CO-I exhibited a lower relative concentration compared with the CO standard, whereas the other commercial CO samples displayed relative ion count values similar to the CO standard (Table 2).

To corroborate the HPTLC PCA groupings, a similar PCA was conducted using LC-MS data (Figure 7) and visualized as scores plot, providing further details on the samples. Figure 7A shows grouping among the CO-standard, CO-I, and CO-J. In contrast, CO-E and CO-F form a distinct cluster, with CO-F showing the greatest divergence (Figure 7A). In Figure 7B, CO-E and CO-F have profiles that are distinct from the edible oils tested, while CO-I and CO-J tightly cluster with them, suggesting possible adulteration (Figure 7B). Overall, most CO’s A, B, C, and D clustered more closely with the CO-standard around the plot origin, reflecting their similarity (Figure 7C).

**Figure 7:**
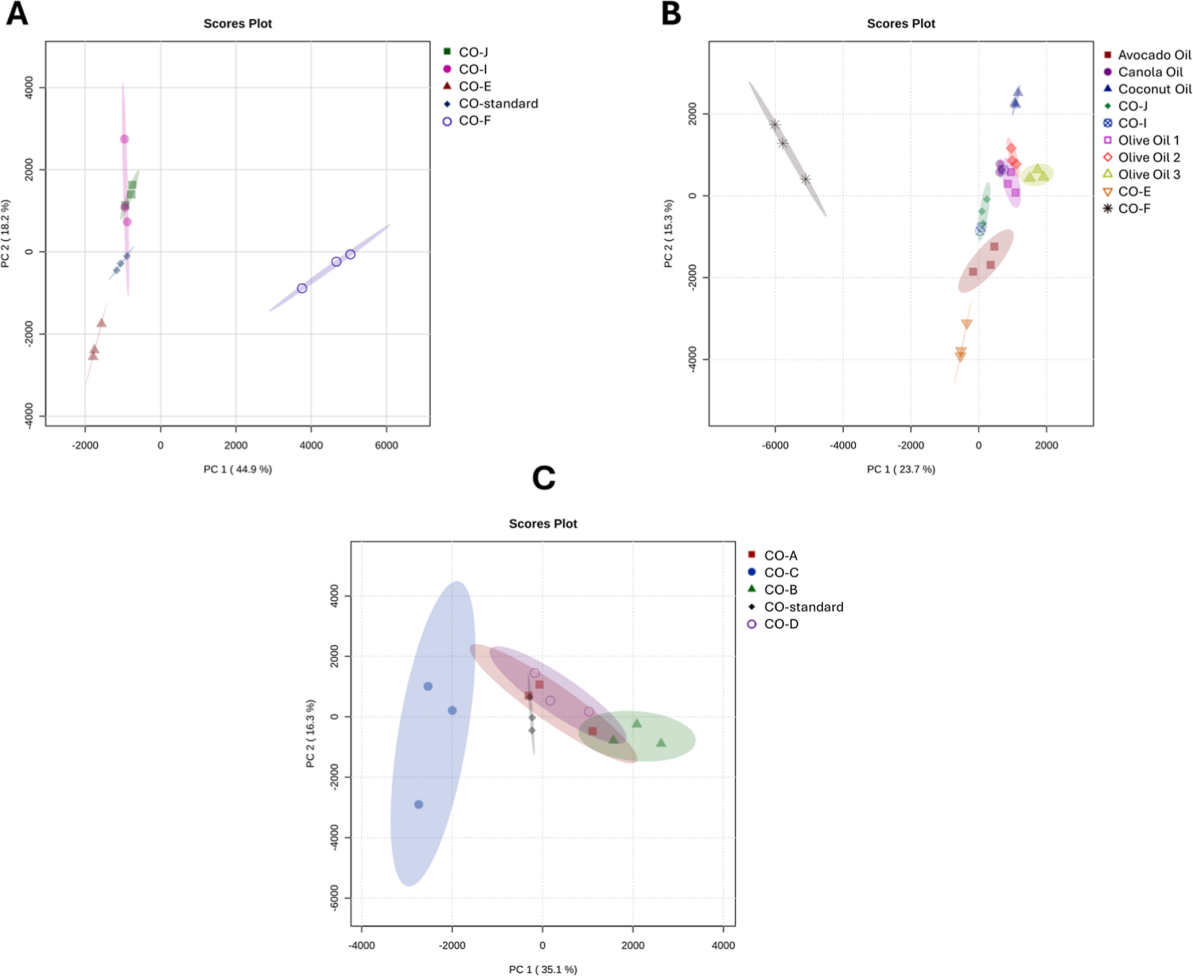
Chemometric PCA based on LC-MS analysis. (A) Comparison between CO-I, CO-J, CO-E, and CO-F with the CO-standard. (B) Evaluation between CO-I, CO-J, CO-E, and CO-F against AVO, CAO, CCO, O1, O2, and O3. (C) Analysis of CO-A, CO-B, CO-C, and CO-D samples against the CO-standard. All samples were analyzed using *n* = 3 replicates.

The PCA plots in Figure 7 present complementary analyses of the samples. Figure 5 highlights that CO-E, CO-F, CO-I, and CO-J all diverge from the CO standard, whereas mainly Figure 7B shows that only CO-I and CO-J resemble canola and avocado oils, with the two atoms of CO-E and CO-F showing no similarity. Together, these results illustrate both chemical divergence from the standard and potential adulteration or substitution in specific samples.

PCA-based score plots have been widely applied as an effective tool to support authenticity verification processes. This statistical technique can be employed by analyzing either specific marker compounds or the complete chemical profile to distinguish differences related to the samples. Recent studies have demonstrated the effectiveness of this approach in detecting adulteration in avocado oil, highlighting PCA as a simple and reliable screening method.^28^ Similarly, PCA has been successfully used to assess the authenticity of olive oil samples. In this case, the methodology was applied to detect adulteration with sunflower oil, with the PCA plot clearly illustrating varying levels of the adulterant across different samples.^29^

## Conclusion

The HPTLC, NMR, and LC-MS screening methods were efficient in classifying the purchased brands and identifying the bioactive compound PMA as it is a critical chemical marker in this high-value natural product. This study revealed that three tested samples lacked PMA in the matrix and that one sample had a relatively lower concentration of this active compound compared to the CO standard. Such variability has direct implications for dermatological product quality, since insufficient levels of PMA may compromise the expected clinical efficacy of chemical peels, while uncontrolled concentrations could raise safety concerns. These findings emphasize the need for careful assessment and quality control of commercial CO brands currently available on the market to guarantee their efficacy and safety, particularly when they are used in chemical peel formulations or other applications. Additionally, the results demonstrate that the combined use of HPTLC, NMR, LC-MS, and multivariate analysis can serve as a powerful tool for monitoring the quality of CO by suppliers and manufacturing industries.

## Material and Methods

### Chemicals

HPTLC analyses were conducted using glacial acetic acid, *tert*-butyl methyl ether, toluene, and methanol HPLC grade and cyclohexane (ACS reagent ≥99%) from Sigma-Aldrich. The LC-MS analysis was performed using formic acid, acetonitrile, and water LC-MS grade from Honeywell. The DMSO-*d*_6_ (99.9%) from Cambridge Isotope Laboratories was used to develop NMR analysis.

### Standards and Samples

The standard croton oil (CO) and PMA standard were purchased from Sigma-Aldrich. Commercial edible oils (avocado, flax, grape, olive, sesame, canola, and coconut) were acquired from local commerce. Additional commercial CO samples were obtained from China, India and USA, and were identified for this study as CO-A, CO-B, CO-C, CO-D, CO-E, CO-F, CO-G, CO-H, CO-I, and CO-J. All commercial samples were systematically analyzed in triplicate with all the tested analytical techniques.

### Sample Dilution

The CO standard, commercial CO samples, and commercial edible oils were each diluted 1:50 in toluene (20 μL of sample and 980 μL of solvent), and the PMA standard was diluted at 200 μg/mL for HPTLC analyses. For LC-MS, the samples were diluted in a system composed of acetonitrile and water (80:20, v:v), until all samples had a final concentration of 0.24 mg/mL. NMR analysis samples were prepared using 20 μL diluted in 145 μL of DMSO-*d*_6_.

### Organoleptic Characteristics

The CO standard and commercial CO samples (*n* = 3) were evaluated for their appearance, color, and odor based on E1627-Standard Practice for Sensory Evaluation of Edible Oils and Fats elaborated by ASTM International.^30^

### High-Performance Thin-Layer Chromatography

The HPTLC analysis was conducted in a HPTLC system (CAMAG), equipped with a TLC-Visualizer 2, Automatic TLC Sampler 4, Automatic Developing Chamber 2, TLC Scanner 4, and TLC Plate Heater III and a Derivatizer. Analysis was run and data were analyzed using *vision* CATS software version 4.1 following the HPTLC parameters described in the USP general chapter ⟨203⟩^31^ using the universal HPTLC mix as a SST.^32^ 2 μL of the samples and standard solution were applied onto the HPTLC plate silica gel 60 F_254_ (*n* = 3). Chromatography was performed using acetic acid, *tert*-butyl methyl ether, and cyclohexane (1:20:20 v/v/v) as the developing solvent. The detection was performed in shortwave UV (254 nm) prior to derivatization and longwave (350 nm broad band) postderivatization. The tracks were scanned at 280 nm using a TLC Scanner 4 set in adsorption mode from 4.9 to 73.1 mm with a scanning speed of 20 mm/s; the slit was set at 5 × 0.2 mm, and data resolution at 100 μm/step. UV–visible spectra of the zones were recorded from 200 to 450 nm. The plates were derivatized with 10% sulfuric acid in methanol (v/v) freshly prepared. The derivatization reagent was sprayed using the Derivatizer with a yellow nozzle at spraying level 3. After derivatization, the plate was heated at 100 °C for 3 min.

### Liquid Chromatography-Mass Spectrometry

The samples (*n* = 3) were analyzed using a Waters I-Class UPLC system coupled with a Waters G2-XS Q-TOF mass spectrometer. Chromatographic separation was achieved on an Acquity UPLC BEH C_18_ column, with 0.1% formic acid in water (v/v) (A) and 0.1% formic acid in acetonitrile (B) as a binary mobile phase. Chromatographic separation was performed by using a linear gradient elution method, beginning with an initial hold at 75% B for 2.0 min. This was followed by a gradient from 75 to 80% over 1.0 min, a hold at 80% B for 1.5 min, a second gradient from 80 to 90% B over 1.0 min, and a hold at 90% B for 1.5 min. The gradient was then increased from 90 to 100% B over 1.0 min, followed by a wash at 100% B for 2.0 min, and finally re-equilibrated to the starting conditions over 1.0 min. The flow rate was 0.45 mL/min, with a 3 μL injection volume and column temperature at 30 °C. The mass spectrometer operated in both positive and negative ionization modes across a *m*/*z* 100–1800 range, with collision energy for the MS^E^ function ramped between 35 and 45 V.

### Nuclear Magnetic Resonance

The ^1^H NMR analyses were recorded on a Bruker Neo NMR spectrometer operating at 500 MHz and equipped with an H/FC/N TCI 5 mm Prodigy CryoProbe using TopSpin (version 4.1.4). Samples (*n* = 3) were prepared in 3 mm NMR tubes with 150 μL of solvent (DMSO-*d*_6_). Spectra were acquired at 25 °C and processed with MestReNova (version 14.0.0). The chemical shifts are given in δ (ppm) and were referenced to residual solvent signals at 2.50 ppm.

### Data Processing, Chemometric, and Statistical Analysis

Peak profiles from the TLC Scanner 4 at 280 nm were exported as csv files using *vision* CATS 4.1. *R*_f_ values and intensity (AU) were uploaded in an algorithm developed with KNIME software, version 5.4.3. to apply *t*-distributed stochastic neighbor embedding unsupervised nonlinear dimensionality reduction to discriminate CO standard sample from edible oils samples applying *k*-means for clustering. Commercial CO samples were also run with this algorithm. NMR acquired data were analyzed with MestReNova (version 15.0.0) and the chemometric PCA was performed on MATLAB (version 9.0). LC-MS/MS^E^ raw data was visualized with MassLynxTM software (version 4.2).

Additionally, all LC-MS data were imported into Progenesis QI^(R)^ V.3.0 (Nonlinear Dynamics, Waters) and normalized to a quality control sample. All data were filtered by retention time (retain features between 2.0 and 7.5 min), max abundance (retain features >100 max abundance), and *p*-value (remove features with *p*-value > 0.05). This data set was exported as a csv file and all features found in solvent blanks were removed using a Python script. Curated data sets were selected from the master list and imported into MetaboAnalyst 6.0 (https://www.metaboanalyst.ca/) to generate PCA plots (Figure 7). These curated data sets were normalized using Pareto scaling.

## Supplementary Material



## Notes

Stacked ^1^H NMR spectra for CO standard and CO-(A–J); and stacked LC-MS spectra for PMA standard, CO standard, and CO-(A–J) (PDF)
