# Supplementary material for: Multiapproach Analysis Combined with Chemometrics for the Authentication of Commercial Oils of Croton tiglium (L.)
Source: ACS Omega. 2025 Oct 30;10(44):53531–9. doi: 10.1021/acsomega.5c09251 (PMC12613127; doi:10.1021/acsomega.5c09251)
Supplement: Supplementary file 1 [file ao5c09251_si_001.pdf]

Multi-approach analysis combined with chemometrics for the authentication of commercial oils of *Croton tiglium* (L.)

Anna Claudia M. O. Capote[1], Msc, Patrícia M. Campos[2], PhD, Wilmer H. Perera[3], PhD, Airton Kist[4], PhD, Wendy K. Strangman[5], PhD, Thomas Williamson[5], PhD, Sarah A. Barr[5], Carlos G. Wambier, PhD[6], Flávio L. Beltrame[1,2], PhD*

[1]State University of Ponta Grossa, Pharmaceutical Science Post-Graduation Program, Ponta Grossa, Parana, Brazil;
[2]State University of Ponta Grossa, Department of Pharmaceutical Sciences, Ponta Grossa, Parana, Brazil;
[3]CAMAG, Scientific Inc., Wilmington, North Carolina, USA.
[4]State University of Ponta Grossa, Department of Mathematics and Statistics, Ponta Grossa, Parana, Brazil;
[5]University of North Carolina Wilmington, Department of Chemistry and Biochemistry, Wilmington, North Carolina, USA;
[6]The Warren Alpert Medical School of Brown University, Department of Dermatology, Providence, Rhode Island, USA.

E-mail addresses and ORCID numbers

Anna Claudia M. O. Capote:	annacapote7@gmail.com	0000-0003-2960-5428
Patrícia M. Campos:	patimazureki@gmail.com	0000-0003-2659-8023
Wilmer H. Perera:	wilmer.perera@camag.com	0000-0003-3607-8638
Airton Kist:	kist@uepg.br	0000-0001-5740-1057
Wendy K. Strangman:	strangmanw@uncw.edu	0000-0002-6911-4909
R. Thomas Williamson:	williaro@gmail.com	0000-0001-7450-3135
Sarah A. Barr:	sab9514@uncw.edu	
Carlos G. Wambier	carlos_wambier@brown.edu	0000-0002-4636-4489
Flávio L. Beltrame:	flaviobeltra@uepg.com	0000-0001-7067-5802

*Address: Av. General Carlos Cavalcanti, Uvaranas, Ponta Grossa- Brazil, 84030-000; email: flaviobeltra@uepg.com and flaviobeltra@gmail.com; +55 42 99601108.

Table of Figures

Figure S1: Stacked ^{1}H NMR Spectra for CO standard and CO- (A-J)...3

Figure S2: Stacked LC-MS Spectra for PMA standard, CO standard, and CO- (A-J)....................4

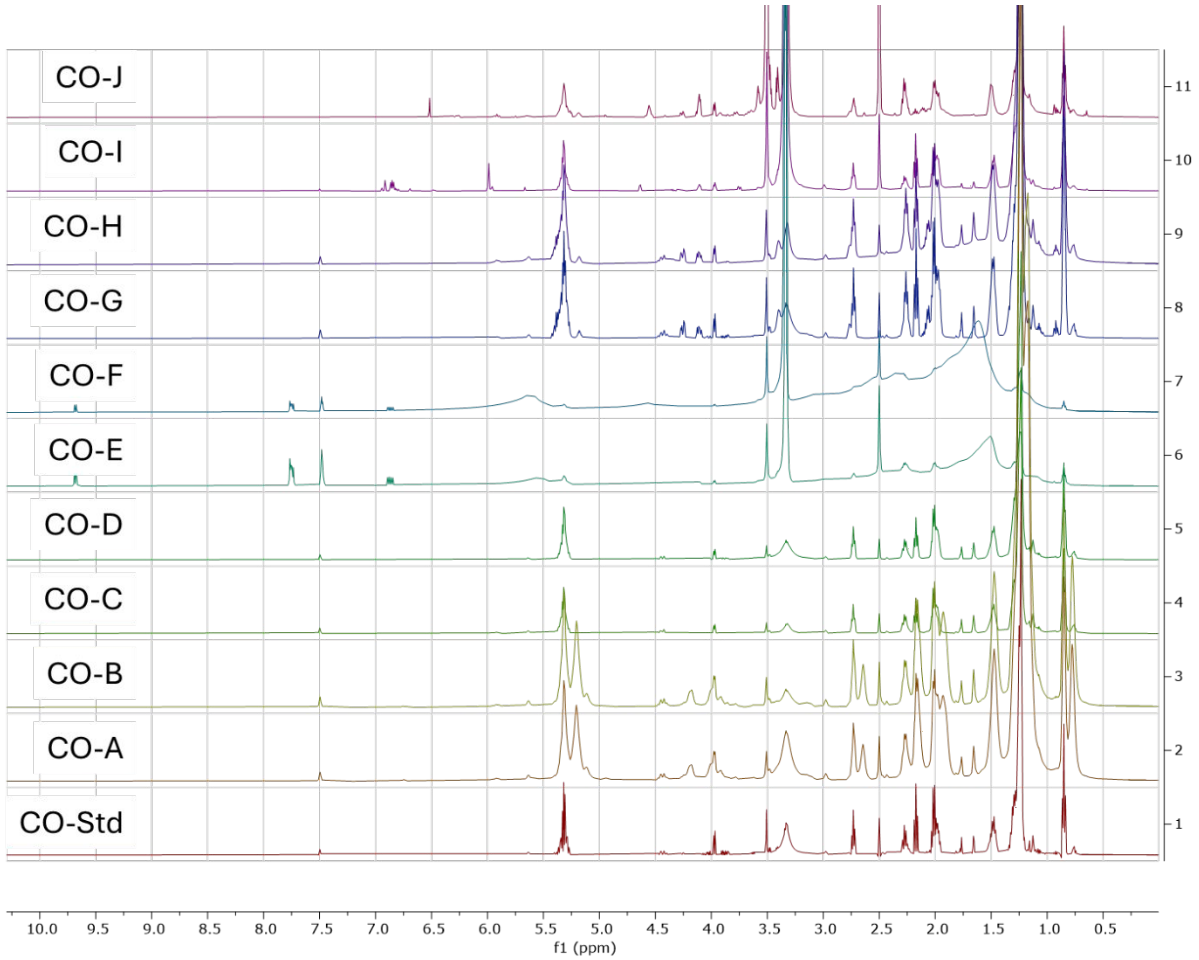

Figure S1. Stacked ^{1}H NMR Spectra for CO standard and CO- (A-J).

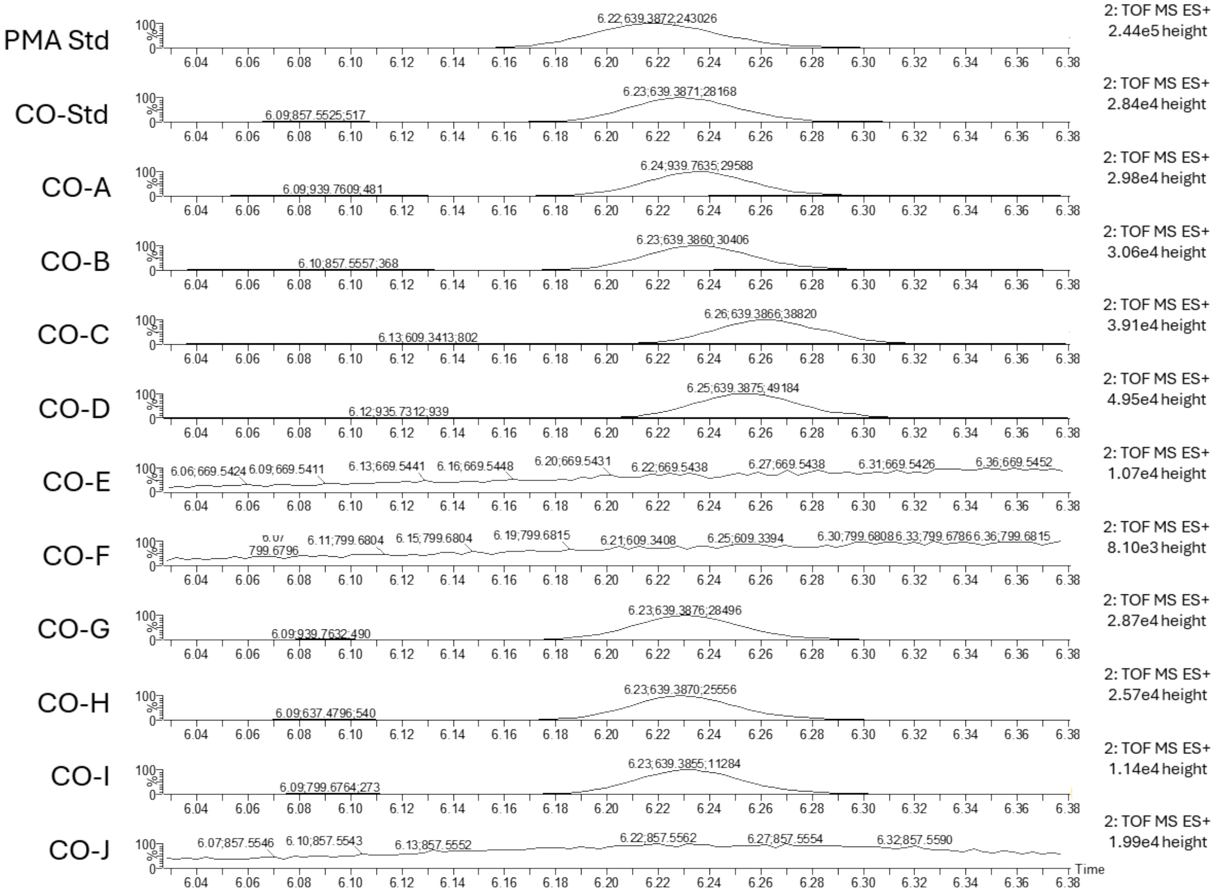

Figure S2. Stacked LC-MS Spectra for PMA standard, CO standard, and CO- (A-J).
